# Indigenous Participation and the Incorporation of Indigenous Knowledge and Perspectives in Global Environmental Governance Forums: a Systematic Review

**DOI:** 10.1007/s00267-021-01566-8

**Published:** 2021-12-02

**Authors:** Melanie Zurba, Anastasia Papadopoulos

**Affiliations:** 1 School for Resource and Environmental Studies (SRES), Dalhousie University, Halifax, NS B3H 4R2 Canada; 2 College of Sustainability, Dalhousie University, Halifax, NS B3H 4R2 Canada; 3 International Union for the Conservation of Nature (IUCN) Commission on Environmental, Economic and Social Policy (CEESP), Gland, Switzerland

**Keywords:** Environmental governance, Global frameworks, Indigenous governance, Global forums, meaningful participation

## Abstract

Global environmental governance (GEG) forums, such as those convened through the United Nations, result in the development of monumental guiding frameworks such as the Sustainable Development Goals (SDGs) and the Convention on Biological Diversity (CBD) Conference of Parties (COPs) Aichi and post-2020 targets. The ratification of policy frameworks by member and/or signatory states can result in major shifts in environmental policy and decision-making and has major implications for Indigenous communities. In this article, we present systematic review of the peer-reviewed literature on Indigenous participation in GEG forums, and focus on the specific questions: (1) what GEG forums include Indigenous participation and (2) how do Indigenous peoples participate in GEG forums, including how their perspectives and knowledges are framed and/or included/excluded within governance discussions, decisions, and negotiations. We provide a bibliometric analysis of the articles and derive seven inductively determined themes from our review: (1) Critical governance forums and decisions; (2) inclusion and exclusion of Indigenous voices and knowledge in GEG forums; (3) capacity barriers; (4) knowledge hierarchies: inclusion, integration, and bridging; (5) representation and grouping of Indigenous peoples in GEG; (6) need for networks among and between Indigenous peoples and other governance actors; and (7) Indigenous peoples influence on GEG decisions and processes. Our findings can be used to improve GEG forums by contributing to the development strategies that address the barriers and inequities to meaningful and beneficial Indigenous participation and can contribute to future research that is focused on understanding the experiences of Indigenous peoples within GEG forums.

## Introduction

Global environmental governance (GEG) forums, such as those convened through the United Nations (UN), result in the development of monumental guiding frameworks such as the Sustainable Development Goals (SDGs)^1^ and the Convention on Biological Diversity (CBD) Conference of Parties (COPs) Aichi^2^ and post-2020^3^ targets. The ratification of policy frameworks by member and/or signatory states can result in major shifts in environmental policy and decision-making and have major implications for communities at the local level. Despite the ongoing and potential for impact from such frameworks on Indigenous peoples, they are often marginalized from environmental decision-making processes or are assigned roles in governance that are less than meaningful and fail to create outcomes that reflect traditional, cultural or spiritual values (Suchet-Pearson et al., 2013; Zurba & Berkes, 2014; Chen et al., 2018). Nevertheless, there is an increasing acknowledgement that Indigenous participation in environmental governance connects to Indigenous rights articulated at the international level through the UN Declaration on the Rights of Indigenous Peoples (UNDRIP), as well as at the regional level through inherent and rights recognition at the level of the state (e.g., Section 35 of Canada’s Constitution affirming Aboriginal treaty rights). Furthermore, there is growing awareness among Indigenous communities, academics, governments, and global conservation organizations that social and environmental benefits should be secured through Indigenous participation and collaboration within GEG systems (Gebara, 2013). Social benefits routinely include employment and enhanced self-esteem; as well as positive emotional and physical benefits that are associated with environmentally based work and engaging in traditional custodial and stewardship responsibilities (Altman & Kerins, 2012). Environmental benefits include enhanced monitoring and stewardship (e.g., fire management) according to traditional knowledge systems (Garnett et al., 2018), and improved community support for managing endangered species (Renwick et al., 2017), among others.

Engagement of Indigenous peoples in GEG forums has been growing alongside the acknowledgement of rights and awareness of the benefits of Indigenous participation in governance (Berkes 2009; Watson et al., 2014; Garnett et al., 2018); however, no assessment of Indigenous participation in GEG forums has taken place to date. Here, we address this issue by conducting a systematic review of the peer-reviewed literature on Indigenous participation in GEG forums, and focus on the specific questions: (1) *what* GEG forums include Indigenous participation and (2) how do Indigenous peoples participate in GEG forums, including how their perspectives and knowledges are framed and/or included/excluded within governance discussions, decisions, and negotiations.

## Methods

The articles collected for our systematic review were found using Scopus, ISI Web of Science and Science Direct research databases. The first two databases were chosen based on their high regard in academic institutions, their comprehensiveness, as well as based on the guidance of Dalhousie University librarians who specialize in approaches to systematic reviews (Bullock & Lawler, 2015; Gjaltema et al., 2020; Klenk et al., 2009; Kivimaa et al., 2016; Liu et al., 2011). Science Direct was included to enhance comprehensiveness of the review overall. In addition, the approach to the review was informed by the Collaboration for Environmental Evidence (CEE) platform which has been used by other scholars to help guide their approaches to and planning for systematic reviews (Bullock & Lawler, 2015; d’Armengol et al., 2018; Malkamaki et al., 2018).

To conduct the review, key search terms were employed using a Boolean search string to filter the results such that they were relevant to the objectives of the paper. The search string employed on the databases included “Indig*,” “Aborig*,” “Native*” in combination with “global environmental governance,” “international governance,” “governance,” “global environment,” and “particip*,” “leadership,” “involv*”. These terms were used to search for peer-reviewed articles that included these search terms in their title, keywords, or abstracts. The authors established inclusion criteria that the papers found in the search results were required to meet in order to be included in the review. The articles were collected and reviewed during the summer of 2020. The papers were required to possess the following characteristics: (1) be peer-reviewed in an academic journal; and (2) refer explicitly to Indigenous participation in GEG. Gray literature was excluded because we aimed to enhance the credibility of our research and outcomes related to the characteristics and quality of participation within GEG forums. The search included papers published from the year 2000 until the summer of 2020 when the review began. Once the papers were collected, the authors employed a snowball approach to acquire further articles using the reference lists of the articles found through the Boolean search on the research databases. The authors read titles, abstracts, and keywords on the papers collected to determine whether they should be included in the review. This allowed the authors to be as comprehensive as possible in the document collection process.

**Fig. 1:**
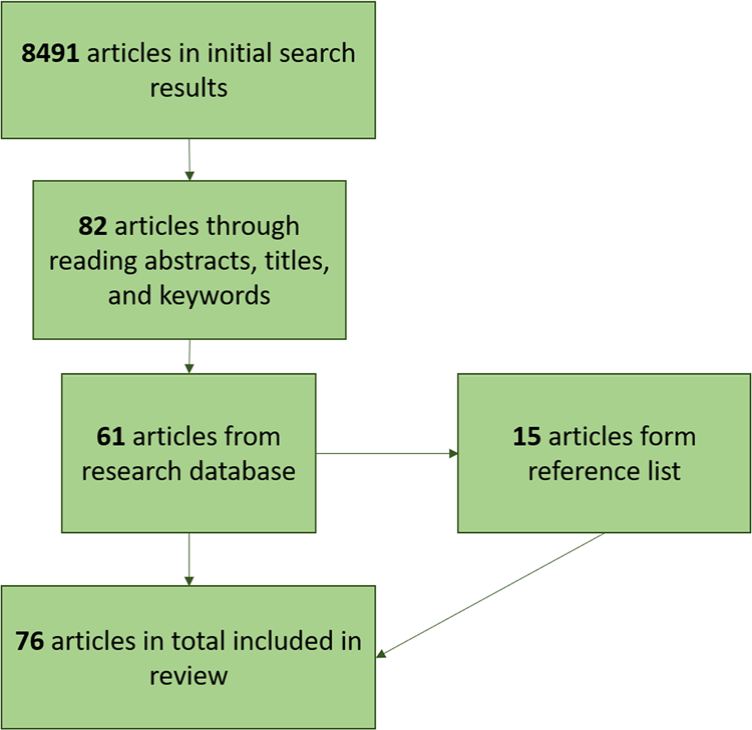
Numbers of articles generated through the systematic review process

After conducting the search on the research databases, a total of 82 articles were collected (Fig. 1). This number was filtered down to 61 articles following an in-depth read of the papers. Some of the articles removed only mentioned Indigenous peoples in a sentence within the paper and did not elaborate or make commentary on their participation in GEG explicitly. Other papers that were removed were centered on Indigenous participation in governance within a particular country whereas this paper sought to explore Indigenous involvement beyond and between national borders. Once these articles were filtered out, an additional 15 articles were found using the reference lists of the articles found through the initial search which brought the total number of peer-reviewed articles to be analyzed to 76. The process for scoping articles for review can be observed in Fig. 1. Once these papers were collected and filtered, they were downloaded into NVivo 10 qualitative data analysis software to be coded deductively and inductively.

The papers were initially coded using a deductive coding framework for basic information which included: (1) year of publication, (2) journal title, (3) author geographical location, (4) geographic location of focus, and (5) keywords. For the papers that had multiple authors, the location of the lead/correspondence author was used. For articles that did not have keywords, the titles of the papers were used to pull keywords for inclusion. Once the papers were coded for their basic information, they were coded using a broad inductive coding framework established by the authors. The coding framework was intended to guide the overarching review process and to allow for coding to occur as themes emerged from the literature reviewed. The coding framework was built around the question of *how* Indigenous participation and involvement in GEG is discussed as well as where (in what forums) these discussions are taking place. When interpreting the spaces where Indigenous participation in GEG is discussed, if a forum was mentioned over ten times in an article, it was coded as being discussed in depth. Furthermore, the “how” of Indigenous participation in GEG was interpreted based on emergent concepts such as how Indigenous peoples are framed within governance discussions, decisions, and negotiations, how Indigenous peoples are (mis)represented, opportunities for participation and influence, and inclusion or exclusion of Indigenous knowledge, perspectives, and experiences in decision-making.

## Results

### Bibliometrics

#### Year of publication

The 76 articles included in this review spanned 14 years—between 2004 and 2020—with the highest number of articles published in 2016 (10 articles) and 2019 (16 articles). The majority of the articles included in this review (57) were published in and after 2014. This increase in the number of articles published is likely due to an increased recognition of the importance of Indigenous peoples’ participation in global governance forums as well as the increase in regional and global networks of Indigenous peoples and organizations that are building capacities to be included in these GEG spaces and recognized as critical environmental governance actors.

#### Journal of publication

There was a wide variety of journals that published articles focusing on the topic of Indigenous participation in GEG forums. There was a total of 48 journals that published papers related to this topic that were found through this review. The most cited journal in this review was *Environmental Science and Policy* which published 6 of the 76 articles included. The journals *Global Environmental Change, WIRES Climate Change*, and *Forest Policy & Economics* each published three articles included in the review. The remaining journals published only one or two of the articles included.

#### Author geographical location

The location of the lead or primary authors of the journal articles included in this review varied, spanning across 18 countries around the world. Most authors were situated in Canada (17), the USA (16), and the UK (13). The remaining authors were dispersed in relatively low numbers (between 1 and 5) among the remaining 15 countries.

#### Geographic area of focus

Most of the papers included in the review (65) focused on one or more global governance forum that was not centered around a particular region or country but were instead discussing GEG more broadly (e.g., the International Union for the Conservation of Nature [IUCN], the COPs, etc.). The next highest area of focus was the Arctic with 3 papers centered around Indigenous participation in Arctic governance. The remaining articles linked regional governance to GEG forums and were geographically focused on the Global South, the Pacific Islands, and Canada/the United States of America. This in part was a result of the fact that articles were filtered for their linkage to GEG forums specifically, and therefore, articles that focused their topic within particular nations were removed.

#### Keywords

**Fig. 2:**
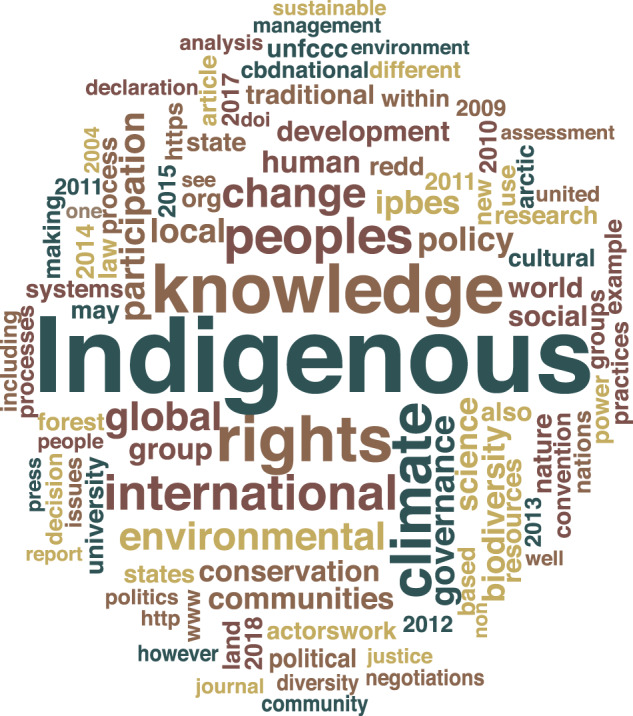
Word cloud of keywords of reviewed articles

There were a very large number of keywords that emerged from the systematic review (Fig. 2). A total of 242 keywords were identified. The three most common keywords used by a significant number of papers include: “Indigenous peoples” (25), “IPBES” (12), and “Indigenous knowledge” (10).

The keywords were also coded for their overall presence (i.e., individual presence rather than collective terms) and “Indigenous,” “governance,” “climate,” “knowledge,” “global,” and “rights,” respectively, were the primary words that were most present overall.

### Emergent Themes from Inductive Review Process

#### Critical governance forums and decisions

**Fig. 3:**
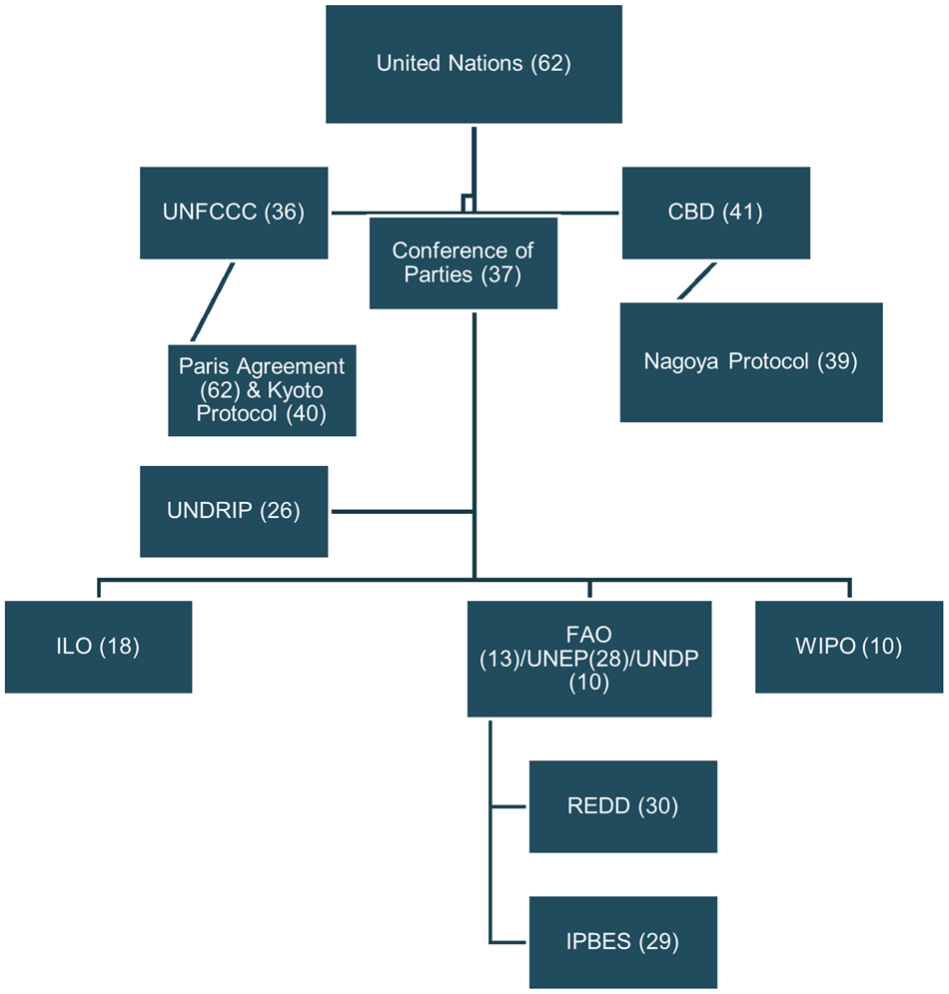
Relationships between GEG forums and number of reviewed articles where they are mentioned

The UN was the primary overarching forum in which global governance activities and Indigenous participation is discussed and documented in the articles included in the review (broadly mentioned in 62 papers) (Fig. 3). The most widely noted influential document for Indigenous participation in GEG that was mentioned in the reviewed papers was the UNDRIP which was adopted and endorsed by the UN in 2007. UNDRIP was mentioned and discussed in 26 articles reviewed. The governance forum under the umbrella of the UN that was mentioned most often was the CBD and associated COPs meetings, articles, working groups and protocols (collectively mentioned 76 times). A total number of 41 articles discussed the CBD overall to some extent. Under the CBD, COP 10 which occurred in Nagoya, Japan, from October 18 to 29 in 2010 was noted as a critical turning point for Indigenous peoples as a central element to this meeting was an emphasis on the importance of Indigenous peoples having autonomy and decision-making authority in the context of the use of genetic resources (e.g., Eimer & Bartels, 2020; Nijar, 2013; Teran, 2016).

The UN Framework Convention for Climate Change (UNFCCC) was the forum that was mentioned the most after the CBD. The UNFCCC was discussed in 36 articles with 20 of the articles addressing the UNFCCC in depth (e.g., Belfer et al., 2019; Wallbott, 2014; Shawoo & Thornton, 2018). Discussions on the UNFCCC were diverse, however, several articles discussed dialog and negotiations around both the UN Reducing Emissions from Deforestation and Forest Degradation (UN-REDD) program and the UNFCCC COPs meetings (e.g., Wallbott, 2014) with mention of both the UNFCCC and UN-REDD in 23 articles. Papers that focused on the UN-REDD program often focused on the inadequacy of Indigenous participation (discussed in a total of 30 papers). The UN-REDD program was established in 2008 through the collaborative efforts of the Food and Agriculture Organization, the UN Environment Programme, and the UN Development Programme. The International Labour Organization (ILO) and the World Intellectual Property Organization (WIPO) were mentioned in 18 and 10 papers, respectively. Mention of the ILO largely focused on Convention No. 169 which articulated Indigenous peoples’ right to retain their customs and traditions (ILO No. 169) as well as Convention No. 120 which articulated the right of Indigenous peoples to self-determination (e.g., Ayani, 2014). Mention of the WIPO largely focused on genetic resources and the right of Indigenous peoples to protect traditional biological knowledge (e.g., Maina, 2011).

The Intergovernmental Science-Policy Platform on Biodiversity and Ecosystem Services (IPBES), which was founded in 2012, was the next most mentioned forum (mentioned in 29 papers). Since this forum was founded most recently, the ratio of mentions within the reviewed articles is quite substantial when compared to the other forums that have been around for much longer. This forum was discussed more deeply in 18 reviewed papers and frequently articulated as a potential space for opening up more opportunities for increased Indigenous participation and knowledge inclusion in environmental governance (e.g., Diaz-Reviriego et al., 2019; Esguerra et al., 2017; Timpte et al., 2018). Figure 3 depicts the relationships between the GEG forums mentioned and the frequency to which they were mentioned in the reviewed articles. In general, all the forums mentioned stem from the UN.

#### Inclusion and exclusion of indigenous voices and knowledge in GEG forums

Even though it is widely agreed that Indigenous participation and knowledge is critical to effective environmental governance, a frequent commentary and position presented from the articles reviewed was that representation of Indigenous peoples is often overlooked or outwardly denied in global governance spaces (e.g., Adeyeye et al., 2019; Belfer et al., 2019). Based on the articles reviewed, several scholars have been commenting on the fact that although recognition is acknowledged as essential, actual inclusion of Indigenous peoples’ voice in environmental governance continues to be limited (Belfer et al., 2019). For example, when it came to signing the UN Framework Convention on Climate Change, the right of Indigenous peoples to participate in discussions was denied (Belfer et al., 2019). There are several barriers articulated in the articles reviewed that sought to unpack the reasons behind the absence and exclusion of Indigenous voice in GEG forums. Firstly, the meaning of active recognition of Indigenous peoples as critical actors in environmental governance is largely undefined or, at the very least, defined in such a way that it can be interpreted vaguely. This can result in participation that is also vague. Furthermore, in spaces where inclusion is “allowed,” it is often the case that the capacity for Indigenous peoples to participate is limited to capacity of the representatives that are attending events, conferences, and negotiations (Belfer et al., 2019; Comberti et al., 2019; Garnett et al., 2018). In many cases, representatives from, for example, governments or large conservation organizations, have the capacity to employ and send several representatives to attend GEG meetings and negotiations to ensure that all the important meetings are attended (Garnett et al., 2018; Larson et al., 2008). However, in many cases, Indigenous peoples’ representatives are often limited in their capacities to take time off work from their own communities and organizations where their participation and leadership in on-the-ground projects and activities are needed (Larson et al., 2008).

There are some governance forums (particularly the REDD program) where Indigenous participation is often critiqued by scholars as not being prioritized in any tangible or meaningful way. The UN-REDD program was one program that was highlighted and often targeted within reviewed papers for its inadequacies of including Indigenous voices. This program is targeted towards supporting national efforts in developing nations to advance emissions reductions. The UN-REDD program has been particularly critiqued as excluding Indigenous voices from discussions, decisions, and establishment of approaches as well as framing Indigenous peoples as victims within international policy (e.g., Ayani, 2014; Dawson et al., 2018; Doolittle, 2010; Eastwood, 2011; Suiseeya, 2017). For example, the UN-REDD program is still being facilitated without significant Indigenous input and REDD projects are still being reviewed by non-Indigenous institutions without the input of Indigenous peoples who are actually being affected by the programs (e.g., Aguilar-Stoen, 2017; Claeys & Pugley, 2016; Shroeder, 2010; Thompson et al., 2011; Wallbott, 2014). This is often the case because the location in which REDD programs are being implemented often overlap with Indigenous territories (e.g., Shroeder, 2010; Shroeder & Gonzalez, 2019).

Despite these overarching perspectives on the exclusion of Indigenous voices in GEG forums, there were some papers that did discuss spaces that have been created to move in the direction of including Indigenous voices in GEG discussions, decisions, meetings, and negotiations. For example, several papers discussed the opportunities that Article 8(j) of the CBD and the Aichi Biodiversity Target 18 initiated. Both sought to address concerns around the exclusion of Indigenous voices. Article 8(j) which states that the signatories to the CBD will: “Subject to national legislation, respect, preserve and maintain knowledge, innovations and practices of Indigenous and local communities embodying traditional lifestyles relevant for the conservation and sustainable use of biological diversity and promote their wider application with the approval and involvement of the holders of such knowledge, innovations and practices and encourage the equitable sharing of the benefits arising from the utilization of such knowledge innovations and practices”. Article 8(j) was mentioned in all 76 articles reviewed and was discussed in depth in 74 of the 76 reviewed papers. Aichi Biodiversity Target 18 which was set out as part of the CBD’s 2010-2020 Biodiversity Goals and supplements Article 8(j) states: “By 2020, the traditional knowledge, innovations and practices of Indigenous and local communities relevant for the conservation and sustainable use of biodiversity, and their customary use of biological resources, are respected, subject to national legislation and relevant international obligations, and fully integrated and reflected in the implementation of the Convention with the full and effective participation of Indigenous and local communities, at all relevant levels” (Decision COP/10/INF/12/Rev.1). Target 18 was mentioned in 73 articles reviewed and discussed in depth in seven of the articles.

#### Capacity barriers

Capacity barriers and other barriers to meaningful participation of Indigenous peoples was a theme that emerged in several of the papers reviewed. These barriers are interconnected and relate to one another; however, they were organized based on some overarching themes through the analysis process (Table 1). In particular, inequities around power and resources were often described as producing both financial and technical constraints that limit Indigenous peoples from being able to participate, access, and navigate environmental governance processes (e.g., Adeyeye et al., 2019; Belfer et al., 2019; Obermeister, 2015).

**Table 1:** Capacity barriers discussed in reviewed papers

Barrier	Type	Explanation	Paper examples
Access	Geography	Situated in isolated areas; limited capacity to access meeting locations	Artelle et al., 2019; Brugnach et al., 2017
	Personnel access	Restricted access to meetings/discussions/negotiations	Belfer et al., 2019; Ciplet, 2014; Maina, 2011; Shroeder, 2010
Resources	Financial capital	Resources to travel and support staff	Ciplet, 2014; Comberti et al., 2019; Corntassel, 2007; Ford et al., 2016b
	Social capital	Number of staff/personnel with skills/experience; translation/language barriers	Aguilar-Stoen, 2017; Adeyeye et al., 2019; Belfer et al., 2019; Ciplet, 2014; Larson et al., 2008; Paulson et al., 2012
	Information	Access to/quantity of information/type of information valued	Adeyeye et al., 2019; Belfer et al., 2019; Corntassel, 2007; Larson et al., 2008; Maina, 2011; Paulson et al., 2012; Teran, 2016; Timpte et al., 2018
Support from external actors	Supportive responsibilities and ensuring appropriate representation	There is no clear indication of which governance actors (e.g., state governments, governance forums, and/or private organizations) should be held responsible for supporting Indigenous participation through distribution of resources for translation, etc.	Belfer et al., 2019; Comberti et al., 2019; Corntassel, 2007, Ford et al., 2016b; Paulson et al., 2012; Shroeder, 2010

With respect to access, there were several papers that articulated the different barriers to accessing environmental governance decision-making processes. Access was largely discussed in the context of: (1) geography and (2) meeting restrictions (Table 1). For example, in the context of geography, several papers noted that many Indigenous peoples are situated in remote and isolated locations that are far from where global environmental decision-making is held and therefore face great barriers to physically travel to GEG meetings which sometimes can lead to an inability to even attend meetings (e.g., Belfer et al., 2019; Brugnach et al., 2017). In the same way that geography limits the ability for Indigenous peoples and organizations to travel to events, and therefore can be both physically and culturally isolated from GEG processes, this barrier also limits opportunities for Indigenous peoples’ representatives to build relationships with other governance actors from different spaces who may be able to offer further support and advocate for Indigenous participation in these meetings (Brugnach et al., 2017). In terms of meeting restrictions, the way in which many GEG meetings are organized (e.g., UNFCCC) result in both open and closed meetings which may require additional steps to be allowed into certain spaces. For example, at the UNFCCC, attendance at some meetings requires the securement of “badges” that require pre-registration through a recognized organization and are limited and distributed via the UN Secretariat (Belfer et al., 2019). This means that at times certain groups are unable to acquire or are deemed as unqualified for receiving such badges. In some cases, Indigenous constituencies are not recognized by the UN Secretariat, therefore, unless they are registered and recognized in advance of the COP meeting, they are not able to gain access to negotiating spaces (Belfer et al., 2019; Kuyper & Backstrand, 2016). Capability is described in some of the articles reviewed as including efforts for enhancing the capacity of groups to influence outcomes (e.g., Ciplet, 2014). As such, to secure attendance at least at the most important meetings, many Indigenous peoples’ representatives have established informal support networks where participants pool and redistribute badges to support those who have missed deadlines and who would be most capable as well as who would most benefit from participating in respective negotiating spaces (Belfer et al., 2019).

Even though in some cases enhanced representation is occurring in GEG spaces, it does not ensure empowerment or agency; lack of capacity can extend beyond the right to be present (Ciplet, 2014). For example, within climate negotiations at the UNFCCC or decision-making in IPBES, there are also some meetings and decision-making processes where Indigenous peoples are only included as “observers” and do not necessarily have the ability to voice their opinions or concerns or ensure their concerns are addressed (Comberti et al., 2019; Ford et al., 2016b; Kovacs & Pataki, 2016; Shawoo & Thornton, 2018). Direct limitation of participation in governance processes can also be observed in governance programs such as UN-REDD where participation and inclusion in activities and on the policy board is determined not based on consensus of affected parties but rather on the UN-REDD Programme Rules of Procedure and Operational Guidance; even if Indigenous peoples are “included” broadly, who is included on the ground is still up to the UN-REDD administration who may be disjointed from the context (Thompson et al., 2011).

In terms of resource capacities, the papers reviewed for this article focused extensively on financial and social capital as well as information-sharing as barriers to participation (Table 1). As mentioned, often times Indigenous peoples are situated in isolated locations which makes accessing and attending GEG meetings, negotiations, and governance processes challenging; however, even beyond the issue of isolation, the financial resources required to reach these meetings serve as an additional layer of complexity for accessing these meetings and events (e.g., Belfer et al., 2019; Ciplet, 2014; Tauli-Corpus et al., 2020; Vierros et al., 2020). These financial constraints often result in Indigenous representatives and leadership from Indigenous Peoples Organizations (IPOs) seeking out funding mechanisms whereby Indigenous peoples’ representatives and organizations are supported to attend GEG events (e.g., Belfer et al., 2019). However, this often results in significant effort and time being dedicated specifically to seek out this funding which further exacerbates the amount of social capital required to even reach these GEG meetings and events as well as limits the capacity of these representatives to prepare for these meetings (e.g., Belfer et al., 2019; Ciplet, 2014). Furthermore, in cases where Indigenous peoples’ representatives acquire the financial resources to reach these meetings, and although representatives of Indigenous peoples and organizations may be “allowed” to participate, the capacities for these groups to attend all important meetings that occur at GEG events is largely dependent upon the resources available to these groups to send an adequate number of personnel to these events (e.g., Larson et al., 2008; Paulson et al., 2012). Many Indigenous communities, nations, and organizations often lack the resources to employ enough people to be sent to all the important meetings held that they have access to. As a result, the onus to attend as many meetings and negotiations as possible is placed on a small pool of individuals representing Indigenous peoples’ and organizations’ interests.

Language is another capacity barrier. Several articles reviewed explain that most discussions at these GEG events occur in English and to add to this, there are increased numbers of acronyms for different working groups, ideas, motions and efforts which make it even more difficult for Indigenous peoples to stay informed when they are facing numerous challenges to simply participate in general, scrambling for funding, staff, and translation services (Belfer et al., 2019; Larson et al., 2008; Paulson et al., 2012; Teran, 2016). Language barriers are often not taken into account—for example, there is often no time dedicated for translation to occur (e.g., Belfer et al., 2019; Comberti et al., 2019; Paulson et al., 2012; Shawoo & Thornton, 2018; Vierros et al., 2020). As such, financial and social capacity deficits can be significant and can hinder attendance, participation, self-representation, and introduce different types of language barriers (Comberti et al., 2019; Paulson et al., 2012; Shawoo & Thornton 2018; Vierros et al., 2020).

To exacerbate these financial and social barriers to accessing and attending GEG events, negotiations, and meetings, access to and capacity to keep up with different information and research emerging from GEG research, projects, and efforts is an additional hurdle that Indigenous peoples’ representatives and IPOs face. On the one hand, there are issues in terms of capacities for Indigenous peoples and IPO representatives to access the breadth of information about GEG approaches and issues in other places because there is disparate and diverse information that is not always easily accessible to all communities. In addition, the type of information that is made accessible is not always necessarily relevant to Indigenous peoples (e.g., the way that the information is shared, produced, disseminated). The final area in which capacity barriers were discussed in the reviewed papers was with references to which governance actors have a responsibility to support Indigenous peoples active and meaningful participation in GEG forums. In recent years, responsibilities for supporting Indigenous peoples’ participation in GEG forums has been informally delegated away from the forums (e.g., UNFCCC) themselves to public or private institutions (e.g., singular donations from governments, such as Norway and Germany to enable Indigenous attendance and support of initiatives such as the Indigenous Pavilion at the UNFCCC) (Belfer et al., 2019). In cases where the GEG forums themselves offer funding, opportunities to receive this support is often specific to particular places (e.g., within nations considered to be “developing”). For example, CBD funding is very specific and often from high-income nations to low-income nations or nations in the Global South even though some advocate that funding through governance forums such as the CBD should support Indigenous peoples regardless of where they are located. Vulnerabilities and marginalization is expressed and occurs in different places in different ways but the CBD funding being very specific and often from high-income nations to low-income nations, limits the chances for Indigenous peoples who may reside in what is deemed a “developed nation” but who do not have access to supports domestically (Ford et al., 2016). Furthermore, to address issues like translation and language barriers, some forums provide and seek out social scientists to conduct translation and in some instances to “represent” Indigenous peoples in negotiations with state actors but this can ultimately result in mistranslation and overrepresentation of “experts” over Indigenous peoples (Diaz-Reviriego et al., 2019; Hill et al., 2020; Tengo et al., 2017).

In some cases, there are arguments that countries should also provide space for Indigenous peoples within those countries to be part of national delegations, however, there are diverse relationships between Indigenous peoples and national leadership; in most cases, few states endorse or support Indigenous peoples attending GEG negotiations (Corntassel, 2007; Maina, 2011). Some nations do not bring the voice of domestic Indigenous peoples to global negotiations (in fact, many do not) and this is directly related to histories and continued marginalization and discrimination by the same states even though they are often looked to as supposedly representing Indigenous peoples (Ayani, 2014; Comberti et al., 2019; Maina, 2011; Paulson et al., 2012). Without providing Indigenous peoples the adequate resources and support to facilitate conservation policies on Indigenous lands, it cannot be expected of these groups to take on the burden of global conservation challenges; it is therefore exceptionally important to promote ‘bottom-up’ approaches to conservation investment and policy design (Garnett et al., 2018).

#### Knowledge hierarchies: inclusion, integration and bridging

Knowledge was another theme that emerged through the systematic review that proved to be a critical element to discussions, debates, and negotiations related to Indigenous peoples and GEG. Knowledge—and in the inclusion, recognition, and influence—of Indigenous knowledge was the focus of several papers. A total of 31 papers discussed the hierarchies of knowledge that are embedded in GEG processes; these discussions often explored the hierarchy between “western” scientific knowledge and Indigenous knowledge where western scientific knowledge is often framed as holding more value than Indigenous knowledge (e.g., Adeyeye et al., 2019; Hughes & Vadrot, 2019; Obermeister, 2018; Parks, 2018). In many cases, Indigenous and local knowledge must compete for legitimacy with and within western systems; GEG organizations privilege “global kinds of knowledge” which is often described as knowledge that can be broadly applied and is decontextualized (Denton, 2017; Ford et al., 2016; Gustafsson et al., 2019; Hill et al., 2020; Hulme, 2010; Obermeister, 2015; Raina & Dey, 2020; Suiseeya, 2014). In this way, localized knowledge held by Indigenous communities can be overlooked for its place-based nature.

Furthermore, Indigenous peoples are still being “invited” into spaces in a way that frames science as more legitimate than Indigenous or other forms of knowledge; furthermore, the mechanisms for sharing knowledge and acknowledging responsibility, relationships, and legitimacy can sometimes be overlooked or lost in translation (Adeyeye et al., 2019). Even though there has been increased recognition of the value and importance of Indigenous knowledge in GEG forums in cases like the IPCC Assessment Report AR4, which articulated that Indigenous knowledge must be included within the development of adaptation and management strategies in order for these strategies to be successful, Brugnach et al. (2017) articulated that what is lacking are rules of production, sharing, acquisition, and ontologies that Indigenous knowledge developed and emerged from.

A common concern discussed in the reviewed papers around knowledge was ways in which Indigenous knowledge is being included, articulating the boundaries between including Indigenous knowledge as supplementary, integrating Indigenous knowledge into decision-making processes and therefore “universalizing” the knowledge, or bridging Indigenous knowledge with western scientific knowledge in a way that keeps it distinct but retaining legitimacy and recognition (e.g., Hill et al., 2020; Hulme, 2010; Kovacs & Pataki, 2016; Tengo & Brondizio, 2014; Obermeister, 2015; Rathwell et al., 2015; Smith & Sharp, 2012). The boundaries between western and Indigenous knowledge continue to be fraught with difficulties that posit the knowledge systems as being “incommensurable” and still requiring the legitimization of Indigenous knowledge through scientific methods of validation and verification (e.g., Gustafsson et al., 2019; Lofmarck & Lidskog, 2017; Kuyper et al., 2017; Obermeister, 2015; Obermeister, 2018; Raina & Dey, 2020; Rathwell et al., 2015; Tengo et al., 2017). With this being said, platforms such as IPBES are striving to apply boundary work^4^ to discover and develop ways to synthesize knowledge coming from different systems in a way that allows for a multiplicity of perspectives, definitions and ways of describing key terms such as biodiversity, and without ignoring distinctions between different knowledge types (e.g., Montana, 2017; Obermeister, 2015; Raina & Dey, 2020; Tengo et al., 2017). In the context of IPBES, boundary work is applied through the creation of boundary objects and through boundary organizations that serve as mechanisms to share and translate knowledge: “Knowledge brokerage is key to foster common understandings, and is supported by use of boundary objects (such as jointly produced maps, pictures or conceptual frameworks)” (Tengo et al., 2017). These boundary objects and organizations serve as tools for co-producing and consolidating knowledge around shared understanding for diverse governance actors involved in this forum.

#### Representation and grouping of indigenous peoples in GEG

The next critical theme that emerged from the review were the ways in which representation and framing of Indigenous peoples is posited within GEG spaces. A total of 29 papers mentioned or discussed how many governance forums describe and frame Indigenous peoples homogeneously or heterogeneously in ways that can both disadvantage and offer advantages to Indigenous peoples (e.g., Anaya, 2004; Ayani, 2014; Belfer et al., 2019; Corntassel, 2007; Ford et al., 2016; Paulson et al., 2012; Thompson et al., 2011). Most of the literature shared the common thought that homogenizing Indigenous peoples into one group (e.g., assuming that they all share the same perspectives, are dealing with the same environmental governance issues, and have the capacities to organize, communicate, and concede stances on topics prior to global governance meetings, discussions, and negotiations) can often lead to governance discussions overlooking the different struggles and aspirations that Indigenous peoples have on the ground (Aguilar-Stoen, 2017; Ayani, 2014). For example, governance systems at national levels can differ dramatically and some Indigenous peoples do not have legal or constitutional imperatives that protect Indigenous rights; therefore, in some cases the need for the UNDRIP to be highlighted at the global level is important (Artelle et al., 2019). Another example offered through the reviewed papers were issues of Indigenous peoples’ homogenized grouping at the global level resulting in conflict between Indigenous groups. The assumption that Indigenous peoples share all the same views, have the same priorities, and concerns results in some instances of competition and conflict between IPOs themselves who may wish to highlight different issues and make different types of demands which can result in those groups with greater capacities to have their voices heard over others with less resources (Belfer et al., 2019; Corntassel, 2007).

Another issue with homogenization occurs with the grouping of terms and frames of Indigenous peoples and local resource users. In some places (e.g., in parts of the African continent), a distinction or identification of Indigeneity is not conducted in the same way, and in some places, distinctions are more severe and more important (Paulson et al., 2012; Thompson et al., 2011). To try to remedy the potential harms caused by framing Indigenous peoples as homogenized groups, diversity among Indigenous peoples is often used as a crutch for not articulating clear prescriptions for protecting Indigenous peoples’ rights; however, in doing this, the importance and integrity of cultural norms that are place-based can be overlooked (Anaya, 2004). Different Indigenous communities and groups may possess diverse governance systems and function through distinct customary operating systems (Artelle et al., 2019). By ignoring these differences, the importance of customary leadership and governance can be overlooked when applying overarching principles established at the global level on the ground (Ayani, 2014). Furthermore, the contexts and challenges facing different groups also shed light on the importance of recognizing that one-size-fits-all approaches do not necessarily work when applied locally because the diversity in concerns and challenges require context-specific solutions (Artelle et al., 2019; Ayana, 2004; Ayani, 2014).

To avoid overlooking the specificities that are essential to understand in order for environmental governance to be equitable and effective, regional organizing occurs among many Indigenous peoples who may face similar challenges, opportunities, and barriers based on their geography (e.g., Aguilar-Stoen, 2017; Powless, 2012; Vizina & Kobei, 2017). Some examples include Indigenous peoples living in forest-rich developing nations where REDD implementation is impacting Indigenous livelihoods and so uniting to advocate for their livelihoods is critical to having a greater impact (Thompson et al., 2011; Wallbott, 2014). On the other hand, there have been ways that Indigenous peoples have leveraged opportunities to homogenize their overarching visions at the global governance scale that were identified in the literature reviewed for this paper. One example provided by Doolittle (2010) was the view of Earth being viewed as a living being and the inherent linkages between peoples and their lands. Most papers that discussed homogenization of Indigenous peoples and their views did not necessarily take a stance on the issue overall; rather, they often focused on the negative implications of homogenization and ignoring place-based perspectives and experiences as well as the opportunities to leverage and organize regionally to emphasize unified voices.

#### Need for networks among and between indigenous peoples and other governance actors

The next critical theme that emerged from the systematic review was the need for various forms of networks among and between Indigenous peoples as well as other governance actors. This need for various types of networks or groups was mentioned in 50 of the 76 papers reviewed (e.g., Aguilar-Stoen, 2017; Belfer et al., 2019; Brosius, 2004; Chen & Gilmore, 2015; Comberti et al., 2019; Hill et al., 2020; Kuyper et al., 2017; Martello, 2008; Oviedo & Pyschkarsky, 2012; Vierros et al., 2020). Transnational organizing and coalitions between Indigenous peoples have opened up space for Indigenous peoples to influence both national, regional, and global agendas and has been identified as critical for developing further influence and agency in GEG decision-making with examples provided in the reviewed papers within UNFCCC and REDD regimes (Adeyeye et al., 2019; Aguilar-Stoen, 2017; Ciplet, 2014; Dawson et al., 2018; Denton, 2017; Doolittle, 2010; Shroeder, 2010; Wallbott, 2014).

Much of the debates in putting forward declarations, producing reports, and establishing agreements at the GEG level are surrounded by issues of semantics (Corntassel, 2007; Eimer & Bartels, 2020; Reimerson, 2013; Shroeder, 2010; Teran, 2016). The reviewed papers offered examples where semantics became an issue in negotiations such as in the drafting of UNDRIP where countries like Canada had issues with the inclusion of “Free, Prior, and Informed Consent” (FPIC) and “right to self-determination” (e.g., Corntassel, 2007; Eastwood, 2011). Other examples include states within the UN system opposing the word “peoples” with an “s” rather than “people” when referring to Indigenous peoples. For example, when the UN Permanent Forum on Indigenous Peoples was established, there were several member states of the UN who were unwilling to support the use of the word “peoples” with an “s” because of the potential implications that this term had for the rights of Indigenous nations to self-determination (Corntassel, 2007). As such, with the institutional fragmentation in recognizing and supporting Indigenous peoples within GEG systems, there is an increased need and space for Indigenous peoples to assert their agency in different ways through coordination and strategic partnership to gain more recognition by countries who are resistant to acknowledging their important role in GEG decision-making (Ayani, 2014). These networks and coalitions have also been identified as also helping to share knowledge across scales and facilitate collaboration and learning (Obermeister, 2015). Through creating networks, there can be consolidation among Indigenous peoples to advocate for particular language that is universally viewed as important such as FPIC.

There are a number of different types of networks formed by Indigenous peoples described in the reviewed papers (Table 2). Predominantly, the papers reviewed focused on networks that have been established both formally (e.g., IIPCC) and informally through regional organization (e.g., the sharing of UNFCCC badges). Indigenous networks create platforms for coordination and redistributing resources and support. Although they are not a permanent solution to addressing institutional power dynamics, they are a mechanism that can enhance opportunities for Indigenous peoples to develop their agency and participation in GEG forums (Belfer et al., 2019). The other critical type of network that was mentioned in several papers were the networks established between Indigenous peoples and NGO or researcher networks to increase their agency. These networks provide a variety of benefits that include advancing shared values for the protection of the environment, support for the development of regional networks and working groups, as well as opportunities to advance the facilitation of Indigenous knowledge inclusion and interaction of different knowledge systems (Aguilar-Stoen, 2017; Suiseeya, 2014; Obermeister, 2015). Eastwood (2011) discussed how although NGOs and civil society organizations may not always have the same objectives, they can come together around shared interests and mobilize shared points of entry for discussions within GEG forums. A few papers discussed the opportunities that are created for advancing consideration for addressing human rights issues through the formation of networks with other equity seeking groups such as women and gender constituencies and other local resource user communities who face similar challenges in having their concerns, knowledge, and experiences included in GEG negotiations and discussions (Belfer et al., 2019; Mercon et al., 2019). Overall, transnational organizing and coalition building among Indigenous peoples, their allies, and other marginalized groups have opened up opportunities to influence GEG agendas from local to global scales (Aguilar-Stoen, 2017).

**Table 2:** Examples of governance networks established by Indigenous peoples within and through GEG forums

Type of network	Outcomes	Examples
Indigenous networks (both informal and formal)	• Sharing information and resources (Ciplet, 2014; Tengo et al., 2017)	• Barcelona World Conservation Congress, Alliances Workshop, “From Chico Mendez to Copenhagen: Learning from Forestry Peoples How to Make REDD Work” (Doolittle, 2010)
	• Shared acknowledgment of dispossession of lands/culture (Ciplet, 2014)	• IPOs pool and redistribute badges to each other for attending UNFCCC meetings (Belfer et al., 2019)
	• Legitimacy (Adeyeye et al., 2019)	• Formation of the International Indigenous Peoples Forum on Climate Change (IIPCC) who determine what will be negotiated for at COPs meetings (Claeys & Pugley (2016)
	• Collaborative lobbying for recognition and inclusion of shared visions, concerns, perspectives, worldviews (Adeyeye et al., 2019; Tengo et al., 2017)	• Inuit Circumpolar Conference (ICC) made up of Inuit from Alaska, Canada, Greenland and Chukotka (Russia) to advocate for Arctic Indigenous populations in response to climate change (Martello, 2008)
Indigenous and other marginalized groups alliances	• Strengthen push for human rights and social justice issues related to environmental governance	• Alliances with women and gender constituencies as well as trade unions (Belfer et al., 2019)
	• Stronger, unified voices	• Alliances with local communities through organizations such as within the IPBES (Mercon et al., 2019; Obermeister, 2015)
Indigenous—NGOs and research networks	• Establishing shared values for advocacy that are in alignment with social and environmental justice (Suiseeya, 2014)	• IUCN TILCEPA and CEESP supporting Indigenous rights groups at the Work Parks Congress in Durban, South Africa in 2003 (Brosius, 2004; Paulson et al., 2012)
	• Boundary and bridging organizations to enhance knowledge inclusion and facilitation of interaction between different knowledge systems and governance actors (Aguilar-Stoen, 2017; Obermeister, 2015)	• IPBES Platform establishing a Task Force for fostering the recognition and inclusion of Indigenous peoples’ and local communities’ knowledge (Mercon et al., 2019; Obermeister, 2015)
		• Alliances with Indigenous social movements and CSOs like the World Rainforest Movement (Obermeister, 2015)

#### Indigenous peoples influence on GEG decisions and processes

The final theme that emerged from the reviewed literature was the evolution of Indigenous peoples’ influence on global GEG decisions and processes. There were 39 papers that discussed this shifting influence. Some of the papers focused on certain specific events or declarations (e.g., UNDRIP, the IUCN Durban Accord) while others discussed general changes over time in certain aspects of environmental governance (e.g., protected areas, intellectual property rights and biological rights, knowledge governance, international law and climate governance). Several papers outline how Indigenous peoples have mobilized different mechanisms of advocacy and assertion of their agency to increase their overall inclusion and participation in GEG over time that have produced shifts in global development agendas, human rights bodies within the UN, and environmental protection and conservation (Aguilar-Stoen, 2017; Ayani, 2014; Mercon et al., 2019).

As mentioned, transnational organizing and coalition building among Indigenous peoples and other groups have opened up opportunities for influence across governance scales that have evolved over time (Aguilar-Stoen, 2017). One example was the 1982 Working Group on Indigenous Populations that emerged within the UN system and led to the establishment of the UNDRIP (Powless, 2012). Another historical example was the UN 1992 Rio Conference including a section within Agenda 21 in Chapter 26 that explicitly prioritized strengthening Indigenous peoples’ efforts for “sustainable development” (Chen & Gilmore, 2015). It was also noted that the first Indigenous groups attended the COP in 1998 and created a constituency in 2001 wherein Indigenous peoples from the Global North attended and made a declaration to include Indigenous rights in the CBD (Claeys & Pugley, 2016; Powless, 2012). It was identified that the ways that Indigenous peoples’ concerns were addressed shifted from ways to “integrate” Indigenous concerns into existing structures (e.g., the ILO Article 107) to focusing on recognizing the rights of Indigenous peoples to self-determination (Ayani, 2014). It was also noted that the increasing influence of Indigenous peoples in GEG forums were responded to in different ways depending on the forum. For example, the IPCC claim to take a neutral stance on how and when Indigenous peoples should be participating in GEG discussions, negotiations, and decision-making whereas forums such as the IPBES were established with a direct focus on asking political questions about things in GEG that make participation and representation for Indigenous peoples challenging (Esguerra et al., 2017; Obermeister, 2018; Kovacs & Pataki, 2016).

Key components of this increased influence focused on ways that certain advancements in influence at the global level have impacted national policy. For example, the IUCN World Parks Congress in Durban, South Africa in 2003 has been widely recognized as a monumental event where Indigenous peoples increased awareness of the impacts of protected areas on Indigenous peoples and initiated a paradigmatic shift in approaches to conservation and area-based conservation that has been adopted and recognized in various nations (Brosius, 2004; Paulson et al., 2012). The UNDRIP, endorsed by the UN officially in 2007, asserted the rights of Indigenous peoples and served as a pivotal point wherein Indigenous rights were to be globally acknowledged and respected (Ayani, 2014). Another example can be observed in the 2010 Declaration of the Rights of Mother Nature that was made at the World People’s Conference on Climate Change and the Rights of Mother Earth in Cochabamba, Bolivia. This declaration has had influence on national implementation of the rights of land in countries like New Zealand and within the Sami Parliament in northern Europe (Boluk et al., 2019; Hulme, 2010). Additional examples were noted in certain regions of the world—especially in the Arctic. For example, the Inuit Circumpolar Conference, was pivotal in advancing international law acknowledging the rights of Indigenous peoples associated with the impacts of climate change within the Arctic Climate Impact Assessment (Powless, 2012; Martello, 2008). Ultimately, there have been notable efforts over time by Indigenous peoples to assert their critical role in GEG and have been increasingly influencing decision-making processes.

## Discussion and Conclusions

The results of our review create an important baseline for understanding Indigenous participation in GEG forums and potential pathways for enhancing the benefits of participation in environmental governance at the global level. Forums convened by the UN and large organizations such as the IUCN have a direct impact on and role to play in how Indigenous peoples can equitably and meaningfully participate in GEG forums and more directly harness benefits from their participation. If such bodies leading the development of environmental frameworks are to live up to international commitments to UNDRIP and support meaningful and beneficial participation of Indigenous peoples in GEG forums, it will be necessary for structural and financial supports to be put into place. Structural supports should focus on diminishing the barriers and outright denial to participation in the development of guiding frameworks, such as was the case with the formation of the UN Framework Convention on Climate Change and participation in the negotiations (Belfer et al., 2019). This type of structural change begins with acknowledging the importance of the contributions made by Indigenous peoples as being equal to the contributions made by others who are deemed to be experts (Diaz-Reviriego et al., 2019; Hill et al., 2020; Tengo et al., 2017).

On the side of the institutions developing frameworks, continued support in the form of structural change will also need to include spaces and processes for amplifying Indigenous voices, as well as spaces and processes that are focused towards enhancing collaboration and accountability (Zurba, 2014). Furthermore, truth-telling and grievance platforms are essential for enhancing collaboration within institutions that have caused wrongdoings to communities through the development of policy frameworks (Zurba, 2014). For example, the UN-REDD Programme could implement such a platform and use CBD Article 8(j) as a guide for promoting the involvement of Indigenous knowledge holders in the development and implementation of frameworks. However, for such platforms to be effective it is important that Indigenous peoples have the capacity to attend. Individual countries, especially developed countries like Canada, also have an important role to play in supporting Indigenous peoples to reach GEG forums (Belfer et al., 2019). It is important in such instances that Indigenous nations and organizations remain in a position to determine who represents them, what their participation looks like and how they might share insights with other communities. Capacity issues relating to participation are not only about attendance or issues that can be supported financially. Enhanced capacity for participation in governance forums also requires supports for overcoming barriers such as language, process exhaustion, as well as a lack of or inadequate information (e.g., briefing materials in the appropriate language and accessible format) being shared prior to participation (Zurba et al., 2016).

Boundary work and boundary organizations have important roles to play in advancing efforts to bring equal value across knowledge systems. Developing focused work around the boundaries that exist within institutions can be highly affective for problem-solving and enhancing collaboration. Boundary work can support marginalized knowledge systems in governance, and lead to knowledge co-creation and the development of shared understandings (Lofmarck & Lidskog, 2017; Zurba et al., 2018). Boundary organizations (a.k.a. bridging organizations) are important actors in governance systems where enhanced participation is required since they there is evidence that they play roles at brokering knowledge, coordinating co-operation, enhancing trust, and managing conflict among desperate groups (Berkes, 2009; Tengo et al., 2017). Regional boundary organizations could play a particularly important role in facilitating and connecting global to Indigenous knowledge, and vice versa. Furthermore, focusing support to regional organizations or branches of GEG bodies creates greater potential for self-determination, self-organization and representative leadership within GEG forums. Increasingly, there is recognition that there is a need to move from Indigenous participation to Indigenous leadership in environmental governance, and that Indigenous-led governance needs to be supported across (sub-national/regional, national, global) scales (Garnett et al., 2018; Artelle et al., 2019).

The findings of our systematic analysis can be used to improve ongoing and future participation in GEG forums by contributing to the development strategies that address the barriers and inequities to meaningful and beneficial Indigenous participation. The themes from our review can also contribute to future research that is focused on understanding the experiences of Indigenous peoples acting within GEG forums and how frameworks emerging from such forums affect Indigenous communities at a local level.

## Footnotes

1 The Sustainable Development Goals are 17 calls to action that emerged from the establishment of the 2030 Agenda for Sustainable Development adopted by the United Nations Member states in 2015. The SDGs offer a “shared blueprint for peace and prosperity of people and the planet” (UN, n.d.).

2 The Aichi Targets were included in Decision X/2 at the tenth COP meeting in 2010 that set out a new Strategic Plan for Biodiversity between 2011 and 2020 for the Parties to the CBD. There are 11 Aichi Biodiversity Targets that were established as a guidepost for implementing the Strategic Plan (CBD, n.d.).

3 The post-2020 targets include 20 action-based targets established at the fifteenth COP meeting in 2020. These targets are included in the Post-2020 Global Biodiversity Framework and the targets are intended to be a guidepost for Parties to the CBD from the years 2021-2030 (CBD, 2020).

4 Boundary work is an approach to problem-solving and research that seeks to break down boundaries that can hinder collaboration amongst diverse groups and support knowledge-sharing, co-creation, and action-oriented outcomes of collaboration (Zurba et al., 2018). Boundary work aims to establish “boundary objects” that are meaningful to all actors involved as a medium for dissolving boundaries of working together across knowledge different knowledge systems on equal footing (Lofmarck & Lidskog, 2017; Zurba et al., 2018)

## References

[CR1] Adeyeye Y, Hagerman S, Pelai R (2019). Seeking procedural equity in global environmental governance: indigenous participation and knowledge politics in forest and landscape restoration debates at the 2016 World Conservation Congress. For Policy Econ.

[CR2] Aguilar-Stoen M (2017). Better safe than sorry? Indigenous peoples, carbon cowboys and the governance of REDD in the Amazon. Forum Dev Stud.

[CR3] Altman JC, Kerins, S (2012) People on country: vital landscapes, Indigenous futures. Federation Press, Sydney, p. 1–22

[CR4] Anaya SJ (2004). International human rights and Indigenous peoples: the move toward the multicultural state. Ariz J Int Comp Law.

[CR5] Artelle KA, Zurba M, Bhattacharyya J, Chan DE, Brown K, Housty J, Moola F (2019). Supporting resurgent Indigenous-led governance: a nascent mechanism for just and effective conservation. Biol Conserv.

[CR6] Ayani I (2014). The dynamics between Indigenous rights and environmental governance: a preliminary analysis and focus on the impact of climate change governance through the Reducing Emissions from Deforestation and Forest Degradation (REDD) programme. Alternative.

[CR7] Belfer E, Ford JD, Maillet M, Araos M, Flynn M (2019). Pursuing an Indigenous platform: exploring opportunities and constraints for Indigenous participation in the UNFCCC. Glob Environ Politics.

[CR8] Berkes F (2009). Evolution of co-management: role of knowledge generation, bridging organizations and social learning. J Environ Manag.

[CR9] Boluk KA, Cavaliere CT, Higgins-Desbiolles F (2019). A critical framework for interrogating the United Nations Sustainable Development Goals 2030 Agenda in tourism. J Sustain Tour.

[CR10] Brosius JP,(2004) Indigenous peoples and protected areas at the World Parks Congress Conserv Biol 18(3):609–612

[CR11] Brugnach M, Craps M, Dewulf A (2017). Including Indigenous peoples in climate change mitigation: addressing issues of scale, knowledge and power. Climatic Change.

[CR12] Bullock R, Lawler J (2015). Community forestry research in Canada: a bibliometric perspective. For Policy Econ.

[CR13] Campbell LM, Corson C, Gray NJ, MacDonald KI, Brosius JP (2014) Studying global environmental meetings to understand global environmental governance: collaborative event ethnography at the tenth Conference of the Parties to the Convention on Biological Diversity. Glob Environ Politics 14(3):1–20. https://muse.jhu.edu/article/552010

[CR14] Chen CW, Gilmore M (2015). Biocultural rights: a new paradigm for protecting natural and cultural resources of Indigenous communities. Int Indigenous Policy J.

[CR15] Chen Y-Shiuan, Suchet-Pearson S, Howitt R (2018). Reframing Indigenous water rights in ‘modern’ Taiwan: reflecting on Tayal experience of colonized common property. Int J Commons.

[CR16] Ciplet D (2014) Contesting climate injustice: transnational advocacy network struggles for rights in UN climate policies. Glob Environ Politics 14(4):75–96. https://muse.jhu.edu/article/558309

[CR17] Claeys P, Pugley DD (2016). Peasant and Indigenous transnational social movements engaging with climate justice. Can J Dev Stud.

[CR18] Comberti C, Thornton TF, Korodimou M, Shea M, Riamit KO (2019). Adaptation and resilience at the margins: addressing Indigenous peoples’ marginalization at international climate negotiations. Environ: Sci Policy Sustain Dev.

[CR19] Convention on Biological Diversity. Strategic plan for biodiversity 2011-2020, including Aichi Biodiversity targets. Convention on Biological Diversity. https://www.cbd.int/sp/

[CR20] Convention on Biological Diversity (2020) Update of the zero draft of the post-2020 global biodiversity framework. Convention on Biological Diversity. https://www.cbd.int/doc/c/3064/749a/0f65ac7f9def86707f4eaefa/post2020-prep-02-01-en.pdf

[CR21] Corntassel J (2007) Partnership in action? Indigenous political mobilization and co-optation during the first UN Indigenous decade (1995-2004). Hum Rights Q 29(1):137–166. https://www.jstor.org/stable/20072791

[CR22] Coscieme L, da Silva Hyldmo H, Fernandez-Llamazares A, Palomo I, Mwampamba TH, Selomane O, Sitas N, Jaureguiberry P, Takahashi Y, Lim M, Barral MP, Farinaci JS, Diaz-Jose J, Ghosh S, Ojino J, Alassaf A, Baatuuwie BN, Balint L, Basher Z, Valle M (2020). Multiple conceptualizations of nature are key to inclusivity and legitimacy in global environmental governance. Environ Sci Policy.

[CR23] d’Armengol L, Castillo MP, Ruiz-Mallen I, Corbera E (2018). A systematic review of co-managed small-scale fisheries: social diversity and adaptive management improve outcomes. Glob Environ Change.

[CR24] Davies K, Adelman S, Grear A, Magallanes CI, Kerns T, Rajan SR (2017). The declaration on human rights and climate change: a new legal tool for global policy change. J Hum Rights Environ.

[CR25] Dawson NM, Mason M, Fisher JA, Mwayafu DM, Dhungana H, Shroeder H, Zeitoun M (2018). Norm entrepreneurs sidestep REDD+ in pursuit of just and sustainable forest governance. Sustainability.

[CR26] Denton A (2017). Voices for environmental action? Analyzing narrative in environmental governance networks in the Pacific Islands. Glob Environ Change.

[CR27] Diaz-Reviriego I, Turnhout E, Beck S (2019). Participation and inclusiveness in the intergovernmental science-policy platform on biodiversity and ecosystem services. Nat Sustain.

[CR28] Doolittle AA (2010). The politics of Indigeneity: Indigenous strategies for inclusion in climate change negotiation. Conserv Soc.

[CR29] Duyck S (2019). Delivering on the Paris promises? Review of the Paris Agreement’s implementing guidelines from a human rights perspective. Clim Law.

[CR30] Eastwood LE (2011). Resisting dispossession: Indigenous peoples, the World Bank and the contested terrain of policy. N Glob Stud.

[CR31] Eimer TR, Bartels T (2020). From consent to consultation: Indigenous rights and the new environmental constitutionalism. Environ Politics.

[CR32] Enns C, Bersaglio B, Kepe T (2014). Indigenous voices and the making of the post-2015 development agenda: the recurring tyranny of participation. Third World Q.

[CR33] Esguerra A, Beck S, Lidskog R (2017). Stakeholder engagement in the making: IPBES legitimization politics. Glob Environ Politics.

[CR34] Ford JD, Cameron L, Rubis J, Maillet M, Nakashima D, Cunsolo Willox A, Pearce T (2016). Including Indigenous knowledge and experience in IPCC assessment reports. Nat Clim Change.

[CR35] Ford J, Maillet M, Pouliot V, Meredith T, Cavanaugh A, IHACC Research Team (2016). Adaptation and Indigenous peoples in the United Nations Framework Convention on Climate Change. Climatic Change.

[CR36] Garnett ST, Burgess ND, Fa JE, Fernandez-Llamazares A, Molnar Z, Robinson CJ, Watson JEM, Zander KK, Austin B, Brondizio ES, Collier NF, Duncan T, Ellis E, Geyle H, Jackson MV, Jonas H, Maler P, McGowan B, Sivongxay A, Leiper I (2018). A spatial overview of the global importance of Indigenous lands for conservation. Nat Sustain.

[CR37] Gebara MF (2013). Importance of local participation in achieving equity in benefit-sharing mechanisms for REDD+: a case study from the Juma Sustainable Development Reserve. Int J Commons.

[CR38] Gjaltema J, Bisebroek R, Termeer K (2020) From government to governance…to meta-governance: a systematic review. Public Manag Rev 22(12):1760–1780. https://doi-org.ezproxy.library.dal.ca/10.1080/14719037.2019.1648697

[CR39] Gonzalez NC, Kroger M (2020). The potential of Amazon Indigenous agroforestry practices and ontologies for rethinking global forest governance. For Policy Econ.

[CR40] Gustafsson KM, Berg M, Lidskog R, Lofmarck E (2019). Intersectional boundary work in socializing new experts. The case of IPBES. Ecosyst People.

[CR41] Harry D (2011). Biocolonialism and Indigenous knowledge in United Nations discourse. Griffith Law Rev.

[CR42] Hill R, Adem C, Alangui WV, Molnar Z, Aumeeruddy-Thomas Y, Bridgewater P, Tengo M, Thaman R, Adou Yao C, Berkes F, Carino J, Carneiro da Cunha M, Diaw MC, Diaz S, Figueroa VE, Fisher J, Hardison P, Ichikawa K, Kairiuki P…, Xue D (2020). Working with Indigenous, local and scientific knowledge in assessments of nature and nature’s linkages with people. Curr Opin Environ Sustain.

[CR43] Hughes H, Vadrot ABM (2019). Weighting the world: IPBES and the struggle over biocultural diversity. Glob Environ Politics.

[CR44] Hulme M (2010). Problems with making and governing global kinds of knowledge. Glob Environ Change.

[CR45] Khan SA (2019). Rebalancing state and Indigenous sovereignties in international law: an Arctic lens on trajectories for global governance. Int Law Pract.

[CR46] Kivimaa P, Hilden M, Huitema D, Jordan A, Newig J (2016). Experiments in climate governance—a systematic review of research on energy and bult environment transitions. J Clean Prod.

[CR47] Klenk NL, Dabros A, Hickey GM (2009). Quantifying the research impact of the Sustainable Forest Management Network in the social sciences: a bibliometric study. Can J For Res..

[CR48] Kovacs EK, Pataki G (2016). The participation of experts and knowledges in the Intergovernmental Platform on Biodiversity and Ecosystem Services (IPBES). Environ Sci Policy.

[CR49] Kuyper JW, Backstrand K (2016). Accountability and representation: nonstate actors in UN climate diplomacy. Glob Environ Politics.

[CR50] Kuyper JW, Linner B, Shroeder H (2017). Non-state actors in hybrid global climate governance: justice, legitimacy, and effectiveness in a post-Paris era. WIRES Clim Change.

[CR51] Larson E, Johnson Z, Murphy M (2008). Emerging Indigenous governance: Ainu rights at the intersection of global norms and domestic institutions. Alternatives: Glob, Local, Political.

[CR52] Liu X, Zhang L, Hong S (2011). Global biodiversity research during 1900-2009: a bibliometric analysis. Biodivers Conserv.

[CR53] Lofmarck E, Lidskog R (2017). Bumping against the boundary: IPBES and the knowledge divide. Environ Sci Policy.

[CR54] MacInnes A, Colchester M, Whitmore A (2017). Free, prior and informed consent: How to rectify the devasting consequences of harmful mining for Indigenous peoples’. Perspect Ecol Conserv.

[CR55] Maina CK (2011). Power relations in the traditional knowledge debate: a critical analysis of forums. Int J Cult Prop.

[CR56] Malkamaki A, D’Amato D, Hogarth NJ, Janninen M, Pirard R, Toppinen A, Zhou W (2018). A systematic review of the socio-economic impacts of large-scale tree plantations, worldwide. Glob Environ Change.

[CR57] Martello ML (2008). Arctic Indigenous peoples as representations and representatives of climate change. Soc Stud Sci.

[CR58] Mercon J, Vetter S, Tengo M, Cocks M, Balvanera P, Rosell JA, Ayala-Orozco B (2019). From local landscapes to international policy: contributions of the biocultural paradigm to global sustainability. Glob Sustain.

[CR59] Montana J (2017). Accommodating consensus and diversity in environmental knowledge production: achieving closure through typologies in IPBES. Environ Sci Policy.

[CR60] Nijar GS (2013). Traditional knowledge systems, international law and national challenges: marginalization or emancipation?. Eur J Int Law.

[CR61] Obermeister N (2018). From dichotomy to duality: addressing interdisciplinary epistemological barriers to inclusive knowledge governance in global environmental assessments. Environ Sci Policy.

[CR62] Obermeister N (2015). Local knowledge, global ambitions: IPBES and the advent of multi-scale models and scenarios. Sustain Sci.

[CR63] Oubenal M, Hrabanski M, Pesche D (2017). IPBES, an inclusive institution? Challenging the integration of stakeholders in a science-policy interface. Ecol Soc.

[CR64] Oviedo G, Pyschkarsky T (2012). World Heritage and rights-based approaches to nature conservation. Int J Herit Stud.

[CR65] Parks L, Shroder M (2018). What we talk about when we talk about ‘local’ participation in international biodiversity law. Open J Sociopolitical Stud.

[CR66] Parks L (2018). Spaces for local voices? A discourse analysis of the decisions of the Convention on Biological Diversity. J Hum Rights Environ.

[CR67] Paulson N, Laudati A, Doolittle A, Welsh-Devine M, Pena P (2012). Indigenous peoples’ participation in global conservation: Looking beyond headdresses and face paint. Environ Values.

[CR68] Powless B (2012). An Indigenous movement to confront climate change. Globalizations.

[CR69] Raina RS, Dey D (2020). How we know biodiversity: institutions and knowledge-policy relationships. Sustain Sci.

[CR70] Rathwell KJ, Armitage D, Berkes F (2015). Bridging knowledge systems to enhance governance of the environmental commons: a typology of settings. Int J Commons.

[CR71] Reimerson E (2013). Between nature and culture: exploring space for Indigenous agency in the Convention on Biological Diversity. Environ Politics.

[CR72] Renwick AR, Robinson CJ, Garnett ST, Leiper I, Possingham HP, Carwardine J (2017). Mapping Indigenous land management for threatened species conservation: an Australian case-study. PLoS ONE.

[CR73] Shawoo Z, Thornton TF (2018). The UN local communities and Indigenous peoples’ platform: a traditional ecological knowledge-based evaluation. WIRES Clim Change.

[CR74] Shroeder H, Gonzalez NC (2019). Bridging knowledge divides: the case of Indigenous ontologies of territoriality and REDD. For Policy Econ.

[CR75] Shroeder H (2010). Agency in international climate negotiations: the case of Indigenous peoples and avoided deforestation. Int Environ Agreem.

[CR76] Smith HA, Sharp K (2012). Indigenous climate knowledges. WIRES Clim Change.

[CR77] Suchet-Pearson S, Wright S, Lloyd K, Burarrwanga L, on the behalf of the Bawaka Country (2013). Caring as country: towards an ontology of co-becoming in natural resources management. Asia Pac View.

[CR78] Suiseeya KRM (2014). Negotiating the Nagoya Protocol: Indigenous demands for justice. Glob Environ Politics.

[CR79] Suiseeya KRM (2017). Contesting justice in global forest governance: the promises and pitfalls of REDD+. Conserv Soc.

[CR80] Tauli-Corpus V, Alcorn J, Molnar A, Healy C, Barrow E (2020). Cornered by PAs: adopting rights-based approaches to enable cost-effective conservation and climate action. World Dev.

[CR81] Tengo M, Brondizio ES (2014). Connecting diverse knowledge systems for enhanced ecosystem governance: the multiple evidence base approach. AMBIO.

[CR82] Tengo M, Hill R, Malmer P, Raymond CM, Spierenburg M, Danielsen F, Elmqvist R, Folke C (2017). Weaving knowledge systems in IPBES, CBD and beyond—lessons learned for sustainability. Curr Opin Environ Sustain.

[CR83] Teran MY (2016). The Nagoya Protocol and Indigenous peoples. Int Indigenous Policy J.

[CR84] Tesar C, Dubois M, Sommerkorn M, Shestakov A (2016). Warming to the subject: the Arctic Council and climate change. Polar J.

[CR85] Thompson MC, Baruah M, Carr ER (2011). Seeing REDD+ as a project of environmental governance. Environ Sci Policy.

[CR86] Timpte M, Montana J, Reuter K, Borie M, Apkes J (2018). Engaging diverse experts in a global environmental assessment: participation in the first work programme of IPBES and opportunities for improvement. Innov: Eur J Soc Sci Res.

[CR87] United Nations Environment Programme (2010) Conference of the Parties to the Convention on Biological Diversity. United Nations Environment Programme. https://www.cbd.int/doc/decisions/cop-10/cop-10-dec-02-en.pdf

[CR88] United Nations General Assembly (2015) United Nations declaration on the rights of Indigenous Peoples: resolution/ adopted by the General Assembly. United Nations General Assembly. https://www.un.org/esa/socdev/unpfii/documents/DRIPS_en.pdf

[CR89] United Nations Department of Economic and Social Affairs The 17 goals (2020) United Nations. https://sdgs.un.org/goals

[CR90] Vierros MK, Harrison A-L, Sloat MR, Crespo GO, Moore JW, Dunn DC, Ota Y, Cisneros-Montemayor AM, Shillinger GL, Watson TK, Goban H (2020). Considering Indigenous peoples and local communities in governance of the global ocean commons. Mar Policy.

[CR91] Vizina Y, Kobei D (2017). Indigenous peoples and sustainable wildlife management in the global era. Unasylva.

[CR92] Wallbott L (2014). Indigenous peoples in UN REDD+ negotiations: “importing power” and lobbying for rights through discursive interplay management. Ecol Soc.

[CR93] Watson JE, Dudley N, Segan DB, Hockings M (2014). The performance and potential of protected areas. Nature.

[CR94] Witter R, Suiseeya KRM, Gruby RL, Hitchner S, Maclin EM, Bourque M, Brosius JP (2015). Moments of influence in global environmental governance. Environ Politics.

[CR95] Zurba M (2014). Leveling the playing field: fostering collaborative governance towards on-going reconciliation. Environ Policy Gov.

[CR96] Zurba M, Berkes F (2014). Caring for country through participatory art: creating a boundary object for communicating Indigenous knowledge and values. Local Environ.

[CR97] Zurba M, Diduck AP, Sinclair AJ (2016). First nations and industry collaboration for forest governance in northwestern Ontario, Canada. For Policy Econ.

[CR98] Zurba M, Maclean K, Woodward E, Islam D (2018). Amplifying Indigenous community participation in place-based research through boundary work. Prog Hum Geogr.

